# Beyond Histones: New Substrate Proteins of Lysine Deacetylases in Arabidopsis Nuclei

**DOI:** 10.3389/fpls.2018.00461

**Published:** 2018-04-10

**Authors:** Magdalena Füßl, Ines Lassowskat, Guillaume Née, Minna M. Koskela, Annika Brünje, Priyadarshini Tilak, Jonas Giese, Dario Leister, Paula Mulo, Dirk Schwarzer, Iris Finkemeier

**Affiliations:** ^1^Plant Physiology, Institute of Plant Biology and Biotechnology, University of Münster, Münster, Germany; ^2^Plant Molecular Biology, Department Biology I, Ludwig-Maximilians-University Munich, Munich, Germany; ^3^Molecular Plant Biology, Department of Biochemistry, University of Turku, Turku, Finland; ^4^Interfaculty Institute of Biochemistry, University of Tübingen, Tübingen, Germany

**Keywords:** lysine acetylation, histone deacetylase, acetyltransferase, Arabidopsis, histones, transcription factors

## Abstract

The reversible acetylation of lysine residues is catalyzed by the antagonistic action of lysine acetyltransferases and deacetylases, which can be considered as master regulators of their substrate proteins. Lysine deacetylases, historically referred to as histone deacetylases, have profound functions in regulating stress defenses and development in plants. Lysine acetylation of the N-terminal histone tails promotes gene transcription and decondensation of chromatin, rendering the DNA more accessible to the transcription machinery. In plants, the classical lysine deacetylases from the RPD3/HDA1-family have thus far mainly been studied in the context of their deacetylating activities on histones, and their versatility in molecular activities is still largely unexplored. Here we discuss the potential impact of lysine acetylation on the recently identified nuclear substrate proteins of lysine deacetylases from the Arabidopsis RPD3/HDA1-family. Among the deacetylase substrate proteins, many interesting candidates involved in nuclear protein import, transcriptional regulation, and chromatin remodeling have been identified. These candidate proteins represent key starting points for unraveling new molecular functions of the Arabidopsis lysine deacetylases. Site-directed engineering of lysine acetylation sites on these target proteins might even represent a new approach for optimizing plant growth under climate change conditions.

## Introduction

Lysine acetylation was first discovered on histones (Allfrey et al., 1964); and the deacetylases and acetyltransferases were originally named accordingly (Brownell et al., 1996; Taunton et al., 1996). Lysine deacetylases (KDACs), formerly referred to as histone deacetylases (HDACs), have profound functions in plant development and acclimation toward abiotic and biotic stresses (reviewed in Luo et al., 2017). KDACs catalyze the removal of the acetyl group on lysine residues, and therefore antagonize lysine acetyltransferases (KATs). While inactivation of some plant KDACs can improve abiotic stress tolerance, the inactivation of others decreases stress resistance and induces developmental defects (Kim et al., 2017; Ueda et al., 2017). The different roles of KDACs partially correlate with their classification into three families: (1) Reduced Potassium Dependency 3/Histone Deacetylase 1 (RPD3/HDA1)-like, (2) the plant-specific type 2 Histone Deacetylases (HD-tuins), and (3) Silent Information Regulator 2-like (sirtuins). The RPD3/HDA1-family, which is referred to as the ‘classical’ KDAC family (De Ruijter et al., 2003), can be further subdivided into three classes: class I (RPD3-like), class II (HDA1-like), and class IV KDACs. Members of the classical KDAC family are Zn^2+^-dependent enzymes, which cleave the acetyl-group of modified lysine residues hydrolytically. Based on sequence homology, the Arabidopsis genome encodes four class I (HDA 6, 7, 9, 19), five class II (HDA 5, 8, 15, 14, 18), and one class IV (HDA 2) KDAC proteins (Ueda et al., 2017). In addition, two potential class I KDAC proteins (HDA10, 17) lacking an intact catalytic domain are encoded in the Arabidopsis genome. For only a few Arabidopsis KDAC proteins, such as HDA6 and HDA14, specific acetylated target proteins, other than histones, have been identified thus far (Tran et al., 2012; Hao et al., 2016; Hartl et al., 2017). Inhibitors have been successfully used to study KDAC functions in various organisms. The fungal antibiotic trichostatin A (TSA) was the first identified and potent inhibitor of all classical KDAC enzymes. Apicidin, another fungal KDAC inhibitor, has a much higher potency for class I KDACs than for class II KDACs, and is thus regarded as a class I specific inhibitor (Bradner et al., 2010). Both inhibitors have recently been used in two different proteomic approaches to elucidate potential *in vivo* substrates of the classical KDACs in Arabidopsis leaf tissue and human cell culture (Schölz et al., 2015; Hartl et al., 2017). Furthermore, KDAC inhibitors can enhance the resistance to salinity in plants, and in humans they are used in cancer therapy (Gallinari et al., 2007; Ueda et al., 2017). Hence, understanding the molecular function of these inhibitors will be fundamental for therapeutic applications, as well as genetic engineering of crops.

## Nuclear Substrate Proteins of the Classical KDACs in Arabidopsis

Under physiological conditions, lysine residues of proteins are usually positively charged. Loss of the positive charge, as well as the increased length of the lysine side chain upon acetylation, can affect the biological function of proteins, such as enzyme activities, protein-protein, and protein-DNA interactions (Yang and Seto, 2008). For example, lysine acetylation regulates the charge of a basic interface on SUMO proteins, which then controls SUMO-mediated interactions (Ullmann et al., 2012). Hartl et al. (2017) identified 77 putatively nuclear KDAC substrate proteins with increased abundance in lysine acetylation after application of TSA or apicidin to Arabidopsis leaves. While acetylation sites on 25 of those proteins were up-regulated by both inhibitors, 39 and 13 proteins were regulated by either apicidin or TSA, respectively. This indicates that different classes of classical KDACs are active in the nucleus of Arabidopsis leaves. However, further studies will be required to match the protein targets with the respective KDAC. In the following, we will discuss the possible implications of lysine acetylation on the functions of selected nuclear protein substrates important in plant stress physiology and development, which might be either direct or indirect targets of the classical Arabidopsis KDACs (Hartl et al., 2017).

## Histones

**FIGURE 1:**
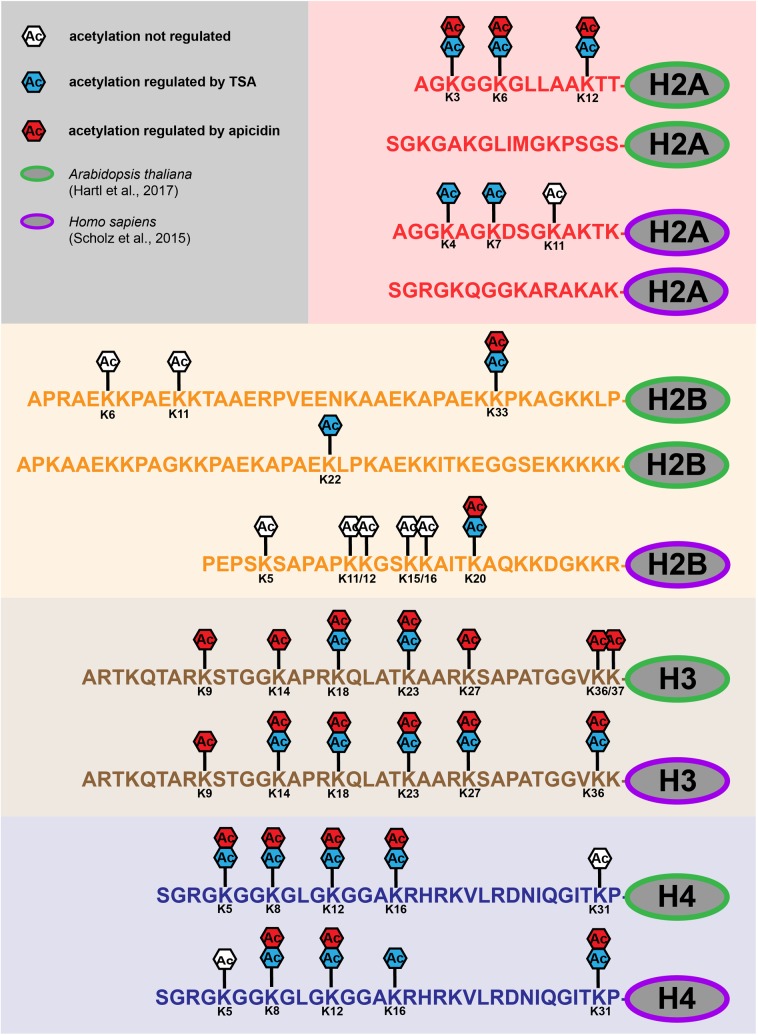
Regulation of lysine acetylation sites of core histones in *Arabidopsis thaliana* and *Homo sapiens*. Up-regulation of lysine acetylation sites (Ac) on histone tails (H2A, H2B, H3 and H4) of Arabidopsis (green) and human (purple) upon TSA (blue) and apicidin (red) treatment. White boxes (Ac) indicate acetylation sites, which were not regulated in abundance upon inhibitor treatments. For details on identifications, see Table EV1a and Table EV2a (Hartl et al., 2017) and Supplementary Table S2 (Schölz et al., 2015). Human histone H2B lysine acetylation sites where derived from multiple proteins differing only a few amino acids in their histone tails (Q93079, B4DLA9, P62807, P33778, P58876, Q8N257, Q99880). Human and Arabidopsis histone H2As and Arabidopsis histone H2B variants exhibit two distinct tails shown here separately. Lysine acetylation sites in structured domains of histones are not depicted.

Histone octamers are responsible for packaging DNA into chromatin. The histone octamers consist of two copies of each H2A-, H2B-, H3-, and H4-type histones (Kornberg, 1974; Luger et al., 1997). The unstructured lysine-rich N-terminal tails of histones (**Figure 1**) are largely conserved in higher eukaryotes (Fuchs et al., 2006; Postberg et al., 2010). At least 20 of these lysine residues of mammalian histones can be acetylated, which is known to stimulate transcriptional activation (Jenuwein and Allis, 2001; Robyr et al., 2004). While the acetylation sites on the H3- and H4- tails are highly conserved between Arabidopsis and human, the sequences of the H2A and H2B-tails are much more diverse (**Figure 1**) (Kawashima et al., 2015). Lysine acetylation sites on all four core-histones were found strongly up-regulated upon KDAC inhibition in plant and human cells (**Figure 1**) (Schölz et al., 2015; Hartl et al., 2017). Acetylation of the histone tails generally results in an open chromatin structure, which makes the DNA more accessible to transcriptional regulators. Acetylated lysine residues are recognized by bromodomains, which serve as acetyl-lysine binding modules (Taverna et al., 2007). Furthermore, lysine acetylation antagonizes other regulatory lysine modifications such as methylation, which modulates transcription by recruiting chromodomain-containing chromatin factors to the DNA template. While several lysine acetylation sites on H3- and H4-type histones have been identified as targets of specific Arabidopsis KDACs previously (reviewed in Luo et al., 2017), the KDAC target sites on H2A- and H2B-type histones were only recently discovered (**Figure 1**) (Hartl et al., 2017). Different H2 variants have important roles in environmental stress acclimation in plants, such as in DNA-strand break repair (Talbert and Henikoff, 2014). Hence, in this context it will be interesting to investigate the specific role of the H2A- and H2B- acetylation sites, and whether all of them are targets of Arabidopsis HDA6 (Earley et al., 2006), or whether other KDACs are also involved in the regulation of H2 acetylation.

## Transcription Factors and Chromatin Remodeling Complexes

**FIGURE 2:**
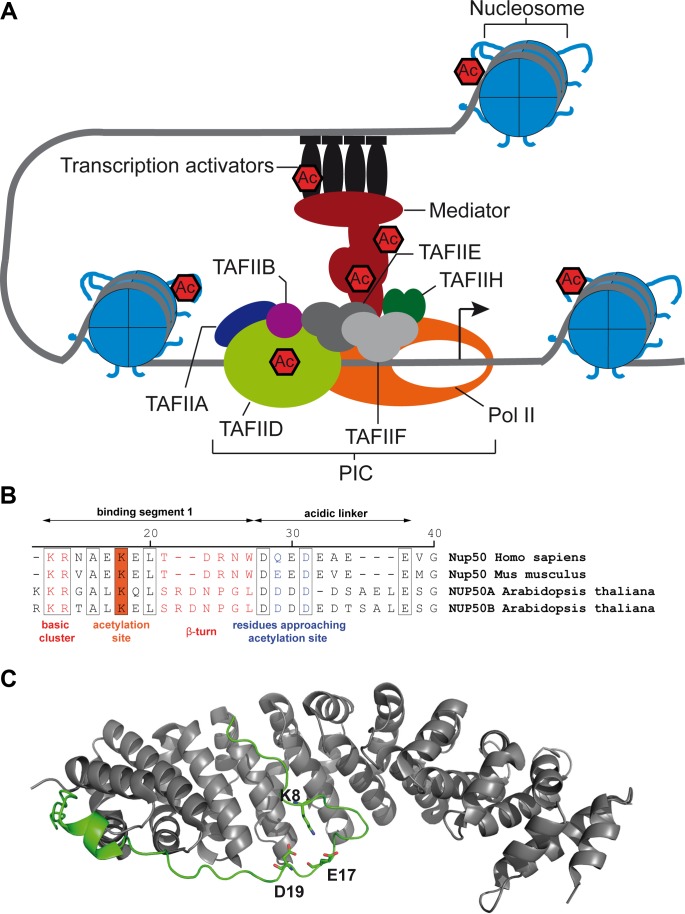
Transcriptional regulators and proteins involved in nuclear import are targets of Arabidopsis RPD3/HDA1-type lysine deacetylases. **(A)** Scheme of the RNA polymerase II-containing preinitiation (PIC) complex with indicated lysine acetylation sites, which were found hyperacetylated upon apicidin treatment in Arabidopsis (Hartl et al., 2017). **(B)** Alignment of Nup50 N-termini of human, mouse and Arabidopsis with indicated lysine acetylation site. Numbers refer to the Arabidopsis sequence. Binding segment 2 is not shown for clarity. **(C)** Structure of murine Nup50-importin-α complex. Importin-α is illustrated in gray, Nup50 in green. K8 and approaching D19 und E17 are illustrated as sticks.

Next to histones, at least 20 different proteins involved in transcriptional regulation have been identified as putative target proteins of the Arabidopsis KDACs (Hartl et al., 2017). It can be expected that these newly identified acetylation sites might have important roles for transcriptional regulation (**Figure 2A**). The zinc finger transcription factor Yin Yang 1 (YY1, At4g06634) becomes hyper-acetylated upon KDAC inhibition (Hartl et al., 2017). YY1 plays important roles in the abscisic acid (ABA) response and was recently identified as regulated by phosphorylation (Wu et al., 2012; Wu and Li, 2017). Hence, it will be interesting to investigate the interplay between phosphorylation and acetylation on YY1. YY1 shows structural similarities with the mammalian YY1 protein, which contains four conserved C2H2 zinc fingers domains. The human YY1 protein is part of the INOSITOL AUXOTROPHY80 (INO80) chromatin-remodeling complex, which enables YY1 to bind its target genes (Cai et al., 2007). Although INO80 is conserved in Arabidopsis, it is still unknown if YY1 is also part of this complex in plants. YY1 can simultaneously act as transcriptional activator and repressor. While it is a negative regulator of ABA-responsive gene expression in plants, it enhances the expression of the ABA REPRESSOR 1 gene and thereby tunes the ABA signaling pathway (Li et al., 2016). In Arabidopsis, YY1 was shown to interact with the protein Mediator 18 (MED18) to suppress the expression of certain disease susceptibility genes, thereby enhancing fungal resistance (Lai et al., 2014). The identified lysine acetylation site (K210) is present in the interaction domain of YY1 and MED18. Hence, the interaction between MED18 and YY1 might be regulated by KDACs to induce the plant resistance against fungal infection. Overexpresison of Arabidopsis HDA19, for example, enhanced the resistance against *Alternaria brassicicola* (Zhou et al., 2005).

In addition to YY1, the transcription factor WRKY1 was identified as potential target of class I KDACs (Hartl et al., 2017). WRKY1, also known as Zinc-dependent Activator Protein-1 (ZAP1), is strongly induced by salicylic acid, and it regulates the plant’s response to pathogen attack (Duan et al., 2007). In addition, it acts as a negative regulator of ABA signaling in guard cells (Qiao et al., 2016). This is especially interesting in the context of HDA6-deficient mutants showing increased drought resistance (Kim et al., 2017). Hence, it will be of great interest to investigate whether drought resistance is mediated via acetylation of WRKY1. The acetylation site within the WRKY 1 (K421) protein lies downstream of the second WRKY domain (AS 301-366) in a unique domain of WRKY1-like proteins.

Another interesting putative KDAC target protein is TIME FOR COFFEE (TIC; AT3G22380). TIC is a nuclear protein required for the light-dependent regulation of the circadian clock in plants (Hall et al., 2003). TIC has been shown to play a role in multiple clock-regulated processes including iron homeostasis (Duc et al., 2009), jasmonic acid signaling (Shin et al., 2012), auxin transport (Hong et al., 2014), and metabolic signaling through SnRK1 to the circadian clock (Shin et al., 2017). Hence, TIC seems to play an important role in synchronizing environmental signals and cellular homeostasis with the circadian clock. *TIC* encodes a nuclear protein without conserved functional domains, rendering sequence-based predictions challenging. TIC potentially contains A-motifs (Ding et al., 2007), typically found in ATP/GTP binding proteins, but this remains to be experimentally verified. Despite its connection to the light-dependent control of circadian rhythm, the expression of TIC and the abundance of the protein remain unaltered throughout the day-night cycle (Ding et al., 2007). This implies that TIC function could be mediated either via PTMs or by interactions with other components. Recently, a lysine acetylation site (K340) was discovered on TIC after TSA treatment (Hartl et al., 2017). Whether this modification bears a significance to the protein-protein interactions and function of TIC needs to be investigated. In this context it is tempting to speculate that acetylation, via the abundance of acetyl-CoA, would function as a signal from cellular metabolism to the circadian clock.

In eukaryotes, transcription starts with the binding of specific transcription factors and chromatin remodelers at gene promoters, which enables the assembly of the preinitiation complex at the core promoter (**Figure 2A**). The preinitiation complex includes RNA polymerase II, as well as the general transcription factors [transcription initiation factor II A (TFIIA), TFIIB, TFIID, TFIIE, TFIIF, and TFIIH], in addition to the Mediator complex. The Mediator complex, composed of at least 25 subunits, is essential for transcription in all higher eukaryotes, and it acts as a transcriptional co-regulator (reviewed in Soutourina, 2017). The Mediator complex directly interacts with various transcription factors, and in plants expression of its subunits is strongly regulated by environmental conditions (Samanta and Thakur, 2015). So far, it is elusive how the protein–protein interactions between Mediator and the different transcription factors, as well as interactions between Mediator subunits, are regulated, but PTMs are thought to play a key role in this (Soutourina, 2017). In Arabidopsis, three subunits of the Mediator complex, MED12 (K930, At4g00450*)*, MED13 (K1676, At1g55325) and MED19a (K210, At5g12230), are hyperacetylated after KDAC inhibition (Hartl et al., 2017). Interestingly, the orthologs of MED12 and MED13 were also identified as KDAC targets in human cell lines (Schölz et al., 2015). In plants, both MED12 and MED13 are known as global regulators of development during germination, vegetative phase change and flowering (Gillmor et al., 2014). Hence, it will be of great interest to investigate whether the developmental defects observed in various KDAC mutants of Arabidopsis are, apart from changes in histone acetylation, also due to altered activities of the Mediator complex (Wang et al., 2014). Additionally, some Mediator subunits, such as MED19a, are also important for abiotic and biotic stress signaling (Seo et al., 2017).

As antagonists of KDACs, KATs are critical for the regulation of gene expression. Reminiscent of kinase activation by auto-phosphorylation, KATs are often activated by auto-acetylation (Karanam et al., 2006). While several KATs were identified as de-acetylation targets of Arabidopsis KDACs, it is interesting to note that KDACs were not identified among the lysine-acetylated proteins. This might indicate that KDACs are regulated by other PTMs, such as nitrosylation (Mengel et al., 2017). Two paralogous acetyltransferases, HAC1 (AT1G79000) and HAC5 (AT3G12980) from the CBP-type family, were identified among the nuclear KDAC substrate proteins in Arabidopsis (Hartl et al., 2017). HAC1 and HAC5 are orthologs of the human p300 and CBP acetyltransferases, respectively, and they have roles in the regulation of flowering time and ethylene signaling (Li et al., 2014). In addition, HAC1 is necessary for resistance against bacteria (Li et al., 2014; Singh et al., 2014). HAC1 and HAC5 showed a marked increase of acetylation on three (HAC1: K1354, K1355, K1359) and four (HAC5: K1330, K1334, K1335, K1339) lysine residues, respectively, after apicidin treatment. All four lysine residues are conserved between both HAC1 and HAC5 and two sites (HAC5: K1330 and K1334) are conserved and found acetylated in the human p300 (K1546 and K1560) and CBP proteins, respectively (Thompson et al., 2004; Schölz et al., 2015). In p300, these sites belong to an auto-inhibitory loop (K1520–1560) that is regulated by auto-acetylation. Removal of this loop constantly activated the acetyltransferase domain (Thompson et al., 2004). A p300 K1560R mutant was defective in acetylation-based enzyme activation. In addition, acetylation of the loop was shown to be under control of KDACs in human cell extracts. Hence, due to the high conservation of the enzyme functions and acetylation sites in the activation loop, it can be expected that the activity of Arabidopsis HAC1 and HAC5 is negatively regulated by KDACs as well. Upon TSA treatment, two additional lysine acetylation sites were identified on HAC1 (K770, K819). The motif and lysine acetylation of K819 is again conserved in human p300 (K1024) and regulated by the sirtuin-type lysine deacetylase SIRT1 (Bouras et al., 2005). In p300, K1024 is part of the cell cycle regulatory domain 1, and is also target of sumoylation. SIRT1 stimulates p300 sumoylation by deacetylating K1024 to suppress p300 activity. Although sumoylation sites have been identified on Arabidopsis KATs (Miller et al., 2013), it is yet unclear whether sumoylation also negatively regulates the activity of HAC1 in Arabidopsis.

GCN5 is the catalytic acetyltransferase subunit of the SAGA (Spt-Ada-Gcn5) complex, which is conserved in eukaryotes and acts as a transcriptional activator at many target loci. In this complex, the subunit Ada2 serves as an anchor to incorporate the GCN5-KAT module into the structure of SAGA (Kassem et al., 2017). In Arabidopsis, three acetylated lysine residues of ADA2a (AT3G07740) were found increased in abundance upon inhibition with apicidin and TSA (Hartl et al., 2017). The three lysine residues, K214, K229 and K230, are located in a protein region of Ada2a, which is involved in binding GCN5 (Mao et al., 2006). Hence, it can be assumed that acetylation of these lysine residues could be a prerequisite for the association of ADA2a with GCN5 (Srivastava et al., 2015). In addition, the TATA-binding protein associated factor 5 (TAF5) becomes hyperacetylated at four consecutive lysine residues (K274, 280, 286, 293) after apicidin treatment. TAF5 is a scaffold protein connecting different structural domains in both SAGA and transcription factor II D (TFIID) complexes (**Figure 2A**). The stretch of acetylated lysine residues in Arabidopsis TAF5 lies within a linker region between two conserved functional domains: the TAF5 N-terminal domain 2 domain (aa 60–200), which defines the homodimer interface and contains a Ca^2+^-binding site, and six WD40 repeat domains (from aa 320–669). WD-repeat domains form a beta-propeller architecture for protein interactions and the WD40 domains of TAF5 were shown to be important for incorporating TAF5 into both TFIID and the SAGA complex in yeast (Durso et al., 2001).

Interestingly, two additional proteins, AtSWC4 (At2g47210) and AtEAF1 (At3g24870), which are thought to be involved in the formation of a nuclear KAT complex NuA4 (Bieluszewski et al., 2015), were identified as putative targets of Arabidopsis KDACs (Hartl et al., 2017). The yeast homolog of SWC4 is part of both the NuA4 complex (and important for histone H4 acetylation) and the chromatin remodeling complex SWR1-C, in which SWC4 is involved in the incorporation of the histone variant H2AZ into nucleosomes (Kobor et al., 2004).

## Nuclear Protein Import: Nup50

Nuclear import of proteins exceeding a mass of 40 kD requires specific carrier proteins (Stewart, 2007). The regulation of nuclear protein import and export is particularly important for the regulation of gene expression during developmental and acclimation responses in all eukaryotes. Different PTMs play key roles in regulating protein import (Christie et al., 2016), and lysine acetylation can modulate the nuclear localization sequences (NLS) of proteins by modulating the residue charge thus altering the interaction with the transport machinery. The most common translocation pathway employs basic NLSs on cargo proteins, importin-β enabling passage through the nuclear pore, and importin-α serving as an adapter between cargo and importin-β. The small GTPase Ran, in its GTP-bound state, disassembles the import complex after transport to the nucleoplasm. In mammals, the cargo release is further facilitated by the Nup50 protein, which binds to the C-terminal region of importin-α and competes for the NLS binding groove (Matsuura and Stewart, 2005). The Arabidopsis genome encodes two nucleoplasm-localized Nup50 homologues, At1g52380 (Nup50a) and At3g52380 (Nup50b), which show significant similarity to mammalian Nup50 (Tamura et al., 2010). Structural analysis of murine Nup50 has revealed that Nup50 interacts with importin-α via two binding segments (Matsuura and Stewart, 2005). Binding segment 2 binds to the C-terminal armadillo repeats of importin-α allowing segment 1 to interact with the NLS binding groove thereby supporting cargo release. Binding segment 1 contains a basic cluster (K3 and R4) that binds to the minor NLS binding site and a β-turn (T11 to W15) that overlaps in part with the major NLS binding site of importin-α (**Figure 2B**). An 8-residue-comprising acidic linker connects both binding segments. Interestingly, binding segment 1 contains a conserved lysine residue (K18), which has been identified as acetylation site (similarly to murine K8) regulated by the KDAC inhibitors apicidin and TSA (Hartl et al., 2017). The crystal structure of the murine importin-α/Nup50 complex shows that K8 approaches an aspartic and a glutamic acid residue in the acidic linker (**Figure 2C**). Acetylation of K18 (AtNup50) or K8 (MmNup50) might hinder the formation of the β-turn that packs against the major NLS binding site of importin-α thereby decelerating the import complex disassembly. A potential modulation of import complex disassembly by lysine acetylation might add a new layer of complexity to the regulation of nuclear transport processes, which might be worthy to investigate.

## Future Directions

The regulation of protein functions by lysine acetylation is evolutionary conserved and has the potential to be highly important for cell signaling and regulation (just like protein phosphorylation). The identification of new substrate proteins of Arabidopsis KDACs now allows to uncover detailed molecular processes in plant development and stress responses, in which the individual classical KDACs are involved in, apart from their deacetylase function on histones. Additionally, further interesting target proteins of other KDAC families such as sirtuins might exist among the lysine-acetylated nuclear proteins that have been recently identified in Arabidopsis (Hartl et al., 2017). One of such example is the lysine acetylation site (K146) on the Arabidopsis cMyc-Binding Protein 1 (AtMBP-1, AT2G36530). It was recently discovered that the stability of AtMBP-1 is regulated by lysine acetylation through the action of the sirtuin-1 deacetylase (Liu et al., 2017). The identified acetylated lysine residues by Hartl et al. (2017) lies in a domain that has previously been shown to be required for MBP1 repressor activity (Subramanian and Miller, 2000). Interestingly, this residue has also been found to be acetylated in human (Choudhary et al., 2009). By investigating the conservation of acetylation sites and regulatory patterns, important key lysine residues can be selected for site-directed mutagenesis in plants. With advances in CRISPR/CAS technologies, these sites are now accessible to study via targeted genome editing. Modifying these lysine residues to constitute acetylated or non-acetylated mimics, might allow a switching of metabolic activities and outputs that can enhance plant yields.

## Author Contributions

MF, IL, GN, MK, AB, PT, JG, DL, PM, DS, and IF performed the data mining, literature mining, sequence analyses, and alignments, and prepared the manuscript. IL, DS, and IF prepared the figures.

## Conflict of Interest Statement

The authors declare that the research was conducted in the absence of any commercial or financial relationships that could be construed as a potential conflict of interest.
